# Spontaneous Acalculous Gallbladder Perforation in an Adolescent Male: A Case Report and Literature Review

**DOI:** 10.7759/cureus.20077

**Published:** 2021-12-01

**Authors:** Hassan Bin Ajmal, Nimra Hasnain, Saima Sagheer

**Affiliations:** 1 General Surgery, Dow Medical College, Dr. Ruth K. M. Pfau Civil Hospital, Karachi, PAK

**Keywords:** acalculous cholecystitis, acute abdomen, type-1 gallbladder perforation, gallbladder perforation, acalculous gallbladder perforation

## Abstract

Though a rare event, gallbladder perforation (GBP) can occur in children and adolescents in the absence of pre-existing cholelithiasis. Moreover, type 1 perforation is seldom found in this cohort. Vague clinical presentation and inconclusive routine investigations can often result in delayed diagnosis. Computed tomography (CT) and intraoperative diagnosis should be utilized to timely diagnose and prevent fatal outcomes. Here, we present a rare case of type 1 GBP in an otherwise healthy 15-year-old boy with no known underlying etiology. Our purpose is to emphasize that although rare, a high clinical suspicion of GBP should be kept in mind even when dealing with acute abdomen in a pediatric population for preventing associated mortality.

## Introduction

With an overall incidence of 2% to 11%, gallbladder perforation (GBP) is extremely rare in adults with potentially life-threatening outcomes. One of the most common causes of GPB is acute calculous cholecystitis [1,2]. Acalculous cholecystitis constitutes only 5% to 10% occurrences, thus making it a highly improbable cause [1].

Literature suggests that children/adolescents are even less susceptible to developing cholecystitis, let alone GBP [3,4]. The fundus is the most common site of GPB due to its blood supply [1,5]. Although GBP is a diagnostic challenge due to its vague clinical presentation, classic hole sign witness on computed tomography (CT) usually aids in making the confirmatory diagnosis [1].

Here, we present a rare case of type 1 GBP in an otherwise healthy 15-year-old boy with no known underlying etiology diagnosed only after exploratory laparotomy. The occurrence of a perforation in an adolescent male together with non-specific symptoms and inconclusive CT findings makes our case highly unique.

## Case presentation

A 15-year-old male school-going adolescent with no known comorbidities was brought to the emergency department with a complaint of acute abdominal pain for one day. The pain was sudden and severe in onset, initially localized to the epigastrium, which after a few hours became generalized. The patient had not passed stool and flatus for one day and had a loss of appetite. There was no associated vomiting and fever. The patient recalled a milder episode of epigastric pain three months ago that resolved on its own. The patient had no history of tuberculosis contact and any febrile illness. Moreover, systemic review and past medical and family history were unremarkable.

On clinical examination, an anxious-looking young boy of average height and build, conscious, and oriented was lying on the bed. Vital signs recorded were heart rate of 95 beats/minute, respiratory rate of 30 breaths/minute, blood pressure of 120/80 mmHg, and he was afebrile. He had a distended abdomen with generalized tenderness more towards the epigastric and umbilical region with marked guarding in the upper part of the abdomen. Gut sounds were absent. On digital rectal examination, the rectum appeared empty and collapsed. The rest of the systemic examinations were unremarkable.

**Figure 1:**
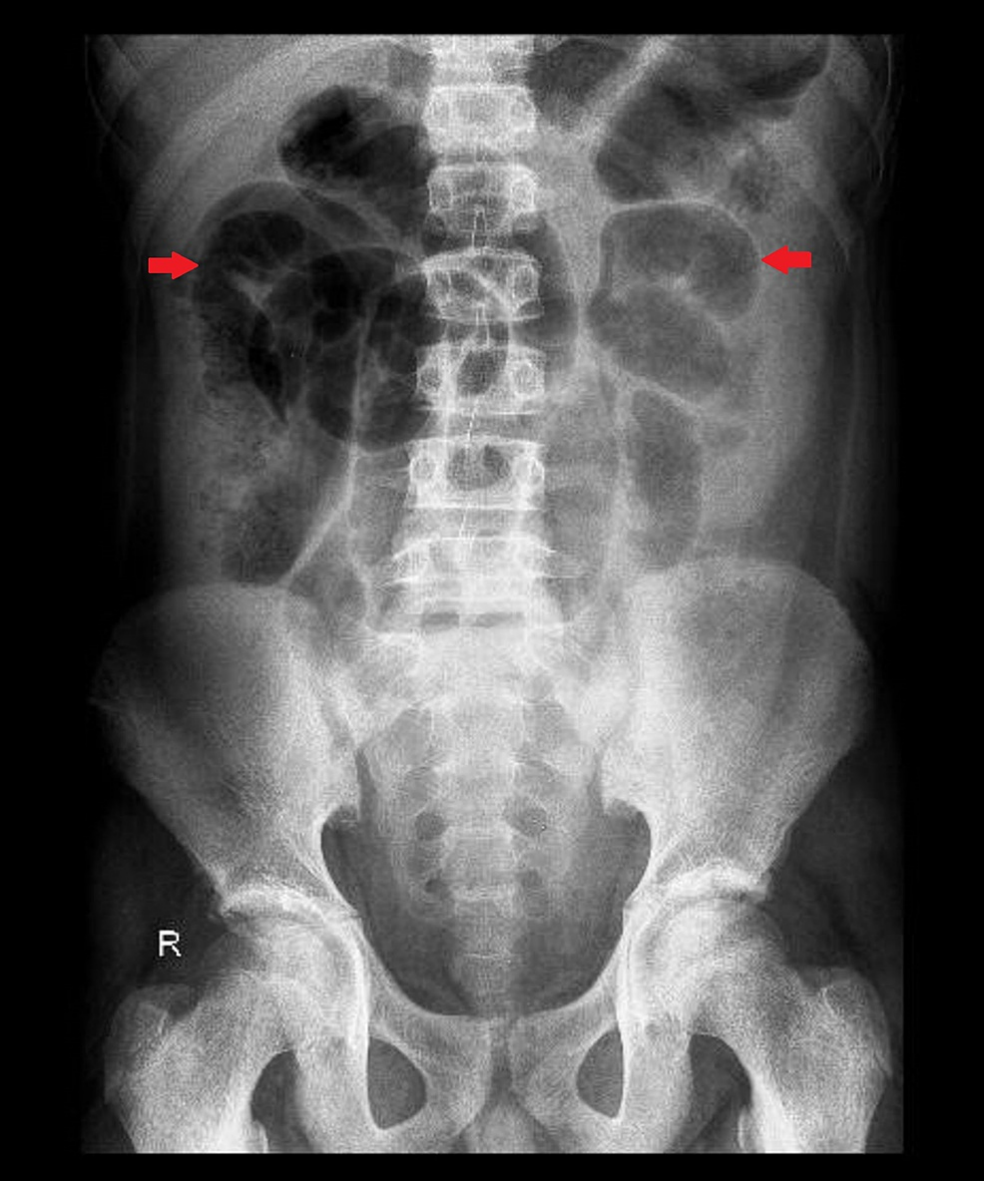
Supine abdominal X-ray showing dilated small bowel loops (arrows).

**Figure 2:**
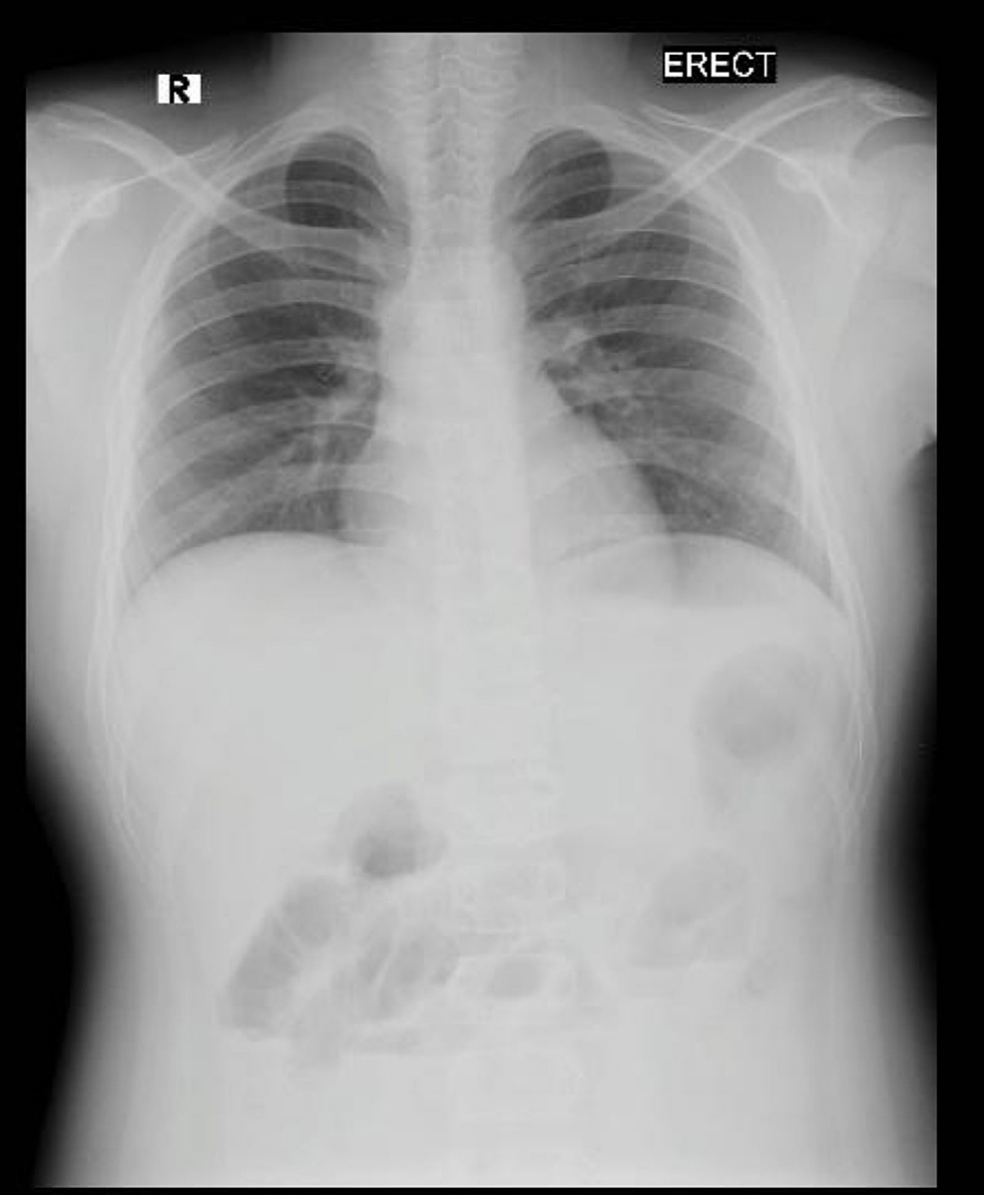
Erect chest X-ray showing normal anatomy.

**Figure 3:**
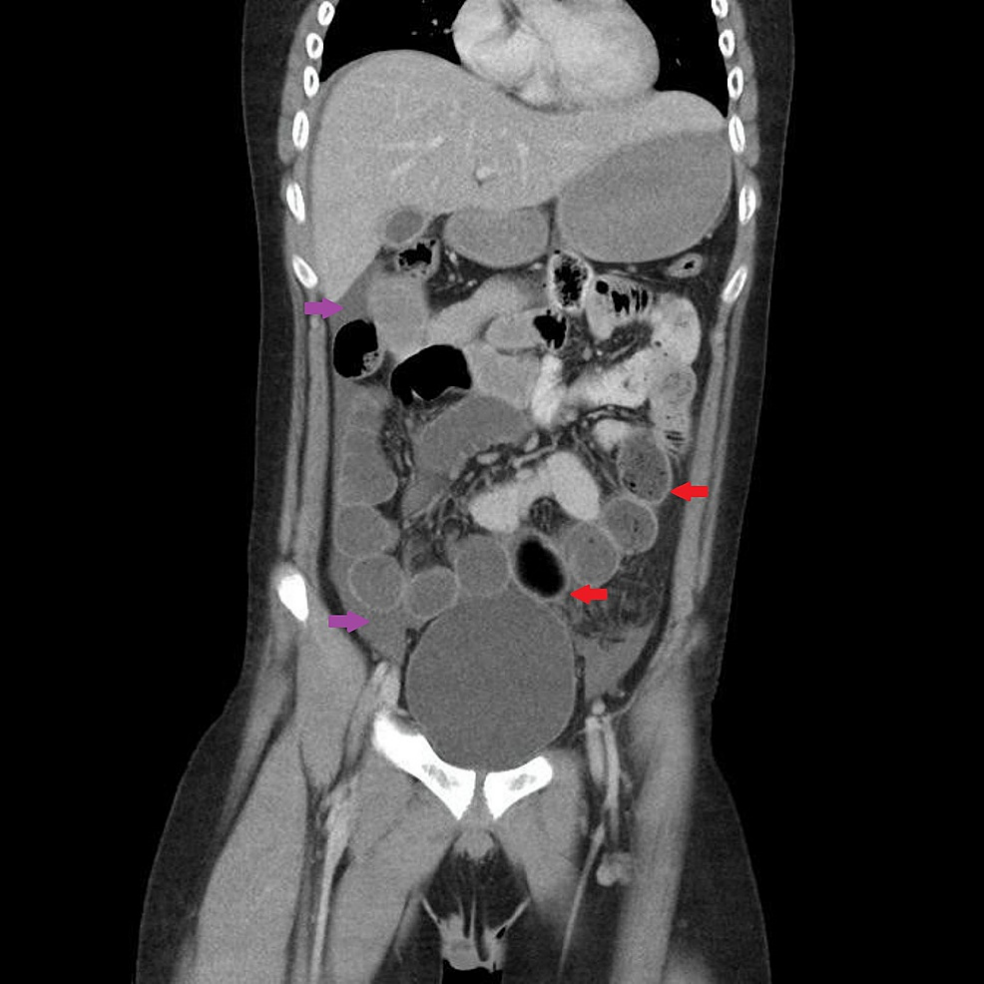
Abdominal computed tomography scan with contrast showing free fluid (purple arrows) around the liver, in the right iliac fossa, and pelvis, with dilated small bowel loops (red arrows).

A provisional diagnosis of acute abdomen with peritonitis was made, with suspicion of a perforated appendix or a hollow viscus perforation. Resuscitation included administration of intravenous (IV) fluids followed by urinary catheterization and placement of a nasogastric tube to decompress the abdominal distension. Laboratory investigations are demonstrated in Table 1. Other laboratory parameters such as liver function tests and urea, creatinine, and electrolytes were normal; viral markers for hepatitis B and C were non-reactive. On radiological inspection, the supine abdominal X-ray (AXR) showed a dilated small bowel with no air in the rectum (Figure 1). The erect chest X-ray (CXR) was, however, unremarkable (Figure 2). The abdominal ultrasonography (USG) revealed a moderate amount of free fluid in the pelvis without any abnormality found in the rest of the scan. The computed tomography (CT) scan with IV contrast of the abdomen showed free fluid around the liver, in the right iliac fossa, and pelvis, with dilated small bowel and no free intraperitoneal air; there was no abnormality in solid organs and hollow viscera (Figure 3).

**Table 1:** Laboratory investigations of the patient. Hb: hemoglobin; RBC: red blood cells; Hct: hematocrit; MCV: mean corpuscular volume; MCH: mean corpuscular hemoglobin; MCHC: mean corpuscular hemoglobin concentration; TLC: total leukocyte count; PLT: platelet; CRP: C-reactive protein; BUN: blood urea nitrogen; ALT: alanine aminotransferase; ALP: alkaline phosphatase.

Laboratory investigation	Patient’s result	Reference range
Hb	13.5	13.0-18.0 g/dl
RBC	4.6	4.5-5.8 million/mcL
Hct	40.1	40-58%
MCV	89	76-96 fL
MCH	30.2	28-32 pg
MCHC	32.9	32-36 g/dL
Neutrophils	88	50-75%
Lymphocytes	7	20-50%
Monocytes	3	1-6%
Eosinophils	2	1-6%
Basophils	0	0-1%
PLT	200,000	150,000-400,000/μL
CRP	56.2	<5 mg/L
Amylase	80	U/L
BUN	18	6-20 mg/dL
Serum creatinine	0.8	0.7-1.6 mg/dL
Bilirubin	0.51	<1.2 mg/dL
ALT	8	7-56 U/L
ALP	108	50-136 U/L
Sodium	138	136-146 mEq/L
Chloride	99	98-106 mEq/L
Potassium	3.7	3.5-5.1 mEq/L

**Figure 4:**
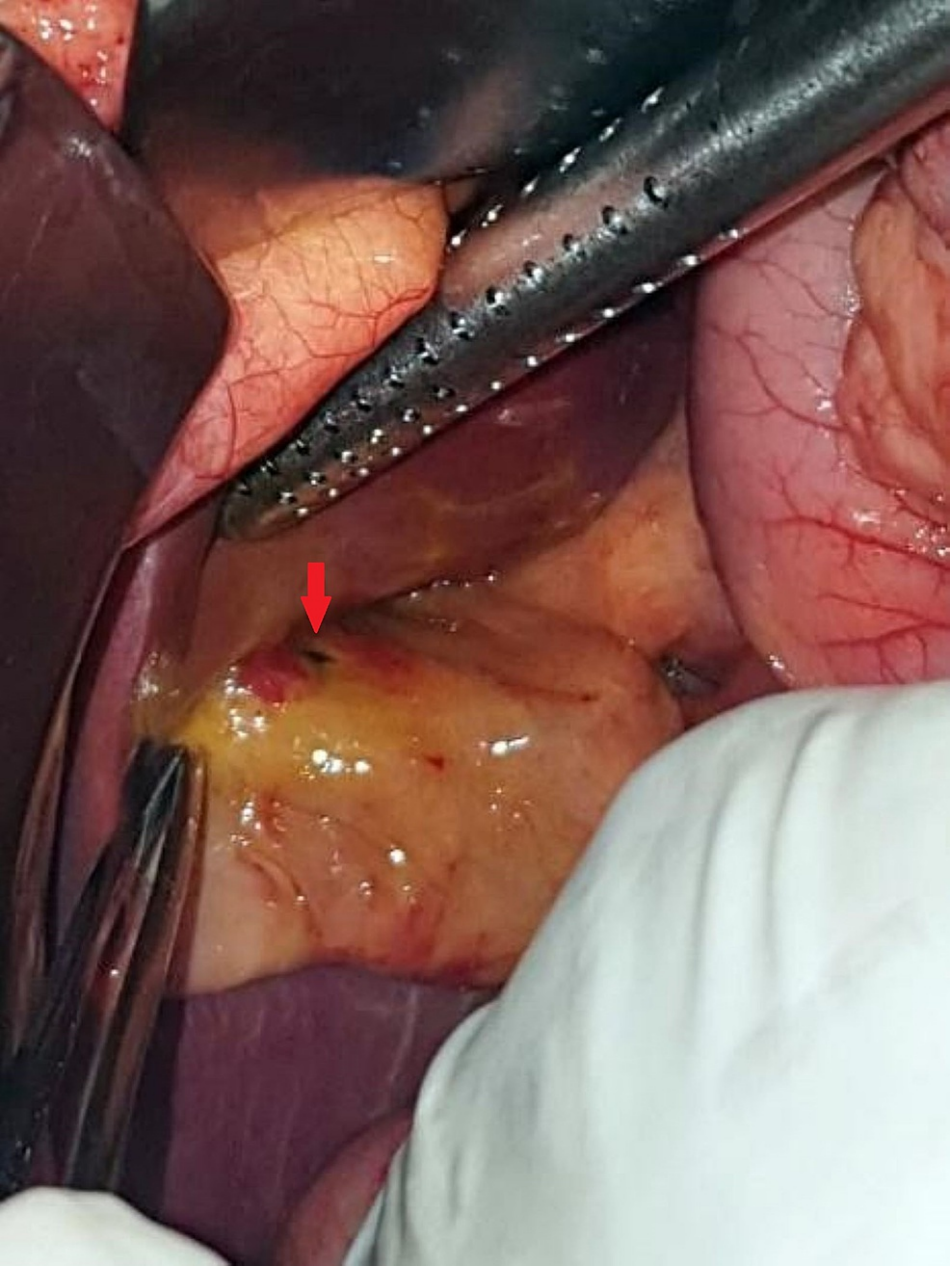
Intraoperative specimen showing a small pinpoint perforation (arrow) of less than 5 mm in the gallbladder.

Subsequently, an exploratory laparotomy with a vertical midline incision was planned. Intraoperative inspection revealed 500 ml of bilious fluid in the peritoneal cavity. The fluid was collected for culture and sensitivity testing. The bowel was run after washing the cavity with warm normal saline. We also found a small pinpoint perforation of less than 5 mm in the gallbladder wall adjacent to the liver (Figure 4); however, there were no stones in the gall bladder. Nevertheless, we performed cholecystectomy. Following the gallbladder removal, the abdomen was cleansed extensively and closed in layers. The gallbladder specimen was sent for histopathology.

The patient had an uneventful recovery postoperatively and was discharged on the fifth postoperative day. Fluid for culture and sensitivity report showed no bacterial growth. The specimen for the histopathology report showed chronic cholecystitis without any evidence of dysplasia or malignancy.

## Discussion

GBP as the complication of acalculous cholecystitis is an infrequent finding, with only a few cases reported among children. Perforations due to enteric fever, systemic stress, i.e. burns, and cardiovascular surgery, and those occurring spontaneously are less common [3,4]. Males are at greater risk of developing GBP, as also seen in our study [2,5].

Chronic cholecystitis is rare in the younger population and presents a different course than in adults. A five-year review of children undergoing laparoscopic cholecystectomy revealed that 87.1% of the population had chronic cholecystitis, though only half of the patients had a chronic symptomatic history [6]. Related studies have reported similar clinical presentations, with most demonstrating acute on chronic cholecystitis. However, the symptoms seemed to be of longer duration, unlike our patient, who had abdominal pain for one day with free perforation and peritonitis [2,7].

Inflammation leading to ischemia and subsequent necrosis is the predominant mechanism behind spontaneous GBP. The fundus is the most susceptible to ischemia since it is the most distal portion of the gallbladder to receive blood. This makes the fundus the most commonly involved site in type 1 GBP [1,5]. According to Niemer, there are three types of GBP; type 1 is acute perforation leading to generalized peritonitis, type 2 is subacute, localized with resultant pericholecystic fluid accumulation, and type 3 is more chronic, followed by a cholecystoenteric fistula formation [8]. The incidence of type 1 and type 2 GBP is widely reported in the literature [9].

GBP is a diagnostic and therapeutic challenge due to its non-specific presentation and smaller perforation size. Often USG and AXR misdiagnose GBP as cholelithiasis and pancreatitis, respectively [1,6,7]. Classic hole sign of GBP is more frequently witnessed on CT than USG, making CT superior to the other routine investigations [1,10]. GBP is often diagnosed using characteristic radiological findings, including wall defect, streaked omentum, pericholecystic fluid accumulation, and gallbladder wall layering. However, acute and non-specific presentation, along with limited radiological resources, led us to make corroborative diagnoses only after exploratory laparotomy [11,12].

Rapid measures to investigate and manage are warranted as GBP is associated with high mortality rates (12-42%) [2,13]. Clinical presentation mimicking acute pancreatitis, cholecystitis, peritonitis, and derangement of laboratory parameters such as raised white blood cell count should be regarded as red flags. Additionally, patients not progressing on expectant management should be investigated rigorously [13,14]. Date et al. reported no difference in the mortality rates with perforation type and age; though, he found that males were associated with higher fatality rates [9]. If not timely treated, complications such as biliary peritonitis, abscess formation in the liver/below the liver, pelvic region, pancreatitis, and possible renal failure may result [15].

To this date, cholecystectomy remains the standard mode of treatment. However, its superiority in terms of safety and efficacy over other therapeutic options, such as percutaneous drainage, is not yet established [9]. Recommended management for type 1 GBP, presenting with acute peritonitis, includes exploratory laparotomy and open cholecystectomy [16]. However, as per existing literature, both type 1 and type 2 perforations have been successfully treated using tube cholecystectomy (percutaneous drainage) without the need for cholecystectomy [7,15]. Additionally, other minimally invasive procedures such as laparoscopic cholecystectomy have been recommended as well [2]. Meanwhile, Garg et al. suggested that cholecystectomy should be indicated only in the presence of gallstones [14]. However, more studies are warranted to compare the efficacy of the different procedures and assess the therapeutic benefit in various perforation types.

## Conclusions

In adolescents/pediatric age groups, a high clinical suspicion of gallbladder perforation is warranted when dealing with acute abdomen, especially when adjunct investigations are not conclusive. Prompt diagnosis and management are crucial in decreasing mortality rates. Radiological investigations may assist in making a presumptive diagnosis, but the definitive diagnosis in most cases is based on intraoperative examination. Adequate follow-up and exploring of the possible causative factors/coexisting etiologies is necessary.
